# Determination of some significant batch culture conditions affecting acetyl-xylan esterase production by *Penicillium notatum *NRRL-1249

**DOI:** 10.1186/1472-6750-11-52

**Published:** 2011-05-16

**Authors:** S Atta, S Ali, MN Akhtar, I Haq

**Affiliations:** 1 Department of Botany, GC University Lahore, Pakistan; 2 Institute of Industrial Biotechnology (IIB), GC University Lahore, Pakistan

## Abstract

**Background:**

Acetyl-xylan esterase (AXE, *EC 3.1.1.72*) hydrolyses acetate group from the linear chain of xylopyranose residues bound by β-1,4-linkage. The enzyme finds commercial applications in bio-bleaching of wood pulp, treating animal feed to increase digestibility, processing food to increase clarification and converting lignocellulosics to feedstock and fuel. In the present study, we report on the production of an extracellular AXE from *Penicillium notatum *NRRL-1249 by solid state fermentation (SSF).

**Results:**

Wheat bran at a level of 10 g (with 4 cm bed height) was optimized as the basal substrate for AXE production. An increase in enzyme activity was observed when 7.5 ml of mineral salt solution (MSS) containing 0.1% KH_2_PO_4_, 0.05% KCl, 0.05% MgSO_4_.7H_2_O, 0.3% NaNO_3_, 0.001% FeSO_4_.2H_2_O and 0.1% (v/w) Tween-80 as an initial moisture content was used. Various nitrogen sources including ammonium sulphate, urea, peptone and yeast extract were compared for enzyme production. Maximal enzyme activity of 760 U/g was accomplished which was found to be highly significant (p ≤ 0.05). A noticeable enhancement in enzyme activity was observed when the process parameters including incubation period (48 h), initial pH (5), 0.2% (w/w) urea as nitrogen source and 0.5% (v/w) Tween-80 as a stimulator were further optimized using a 2-factorial Plackett-Burman design.

**Conclusion:**

From the results it is clear that an overall improvement of more than 35% in terms of net enzyme activity was achieved compared to previously reported studies. This is perhaps the first report dealing with the use of *P. notatum *for AXE production under batch culture SSF. The Plackett-Burman model terms were found highly significant (*HS*), suggesting the potential commercial utility of the culture used (df = 3, LSD = 0.126).

## Background

Xylan is the principle component of plant cell wall hemicelluloses. It is a heteroglycan composed of a linear chain of xylopyranose residues bound by β-1,4-linkage, with a variety of constituents linked to the main chain by glycosidic or ester linkages. The biodegradation of xylan requires a set of esterases and glucanases [1,2]. Among these, acetyl-xylan esterase (AXE, *EC 3.1.1.72*) hydrolyses acetate group from the chain. Together with xylanases, β-xylosidases, α-arabinofuranosidases and α-methylglucuronidase, the enzyme is an integral part of the xylanolytic system that is capable of the complete hydrolysis of xylan. It releases methyl residue from cell wall, degrades cellulose, and liberates acetic acid from *O*-acetyl-galactoglucomannan and *O*-acetyl-4-*O*-methyl-glucuronoxylan [3]. Microbial AXE production has been preferred to plant or animal sources because of the easier availability, structural stability and ease to genetic manipulation. It has been purified and characterized from several microorganisms including bacteria and fungi. In particular, filamentous fungi have demonstrated a great capability for secreting a wide range of xylanases, being the genus *Aspergillus *and *Trichoderma *the most extensively studied amongst the xylan degrading fungi. However, an important amount of information about the production genetics of these enzymes from the genus *Penicillium *has accumulated in recent years. A great number of *Penicillia *are active producers of xylanolytic enzymes, and the use of enzymes from these species has acquired growing importance in biotechnological applications [4]. AXE is a potentially useful enzyme in bio-bleaching of wood pulp, treating animal feed to increase digestibility, processing food to increase clarification and converting lignocellulosic substances to feedstock and fuels [5].

Enzyme biosynthesis depends on the type of the strain, composition of medium, and method of cultivation. Various batch culture techniques have been exploited which include liquid culture and solid sate fermentation (SSF). The later holds a tremendous potential for the production of xylanolytic enzymes. It can be of special interest in those processes where crude preparations may be used directly as an enzyme source [6]. In addition, SSF has a number of merits which include non-aseptic conditions, use of a wide variety of matrices, low energy expenditure, less expensive downstream processing, lower wastewater output, potentially higher reproducibility and easier control of contamination [7]. The organisms need essential elements such as carbon, nitrogen, phosphorus and sulphur for growth and subsequent enzyme production. The pattern of accumulated reducing sugar after specific incubation time is characteristic to each microbial species [3,8]. The utilization of various agricultural by-products as substrate in industrial processes is gaining momentum under batch culture SSF being carried out aseptically. In the present study, wheat bran was exploited as a substrate for AXE production from *Penicillium notatum *NRRL-1249. Various nitrogen sources were tested to improve the overall yield of enzyme. The 2-factorial Plackett-Burman experimental design was used to further identify the significant batch culture conditions influencing AXE production.

## Methods

The chemicals including potassium chloride, magnesium sulphate, sodium nitrate, *p*-nitrophenol and *p*-nitrophenyl-acetate were of analytical grade and purchased from Fluka (UK) and Sigma (USA). All other reagents were of the highest possible purity.

### Organism and Culture Maintenance

*Penicillium notatum *strain NRRL-1249 (initially procured from *Northern Regional Research Labs, Peoria, USA*) was obtained from *IIB Culture Bank*. The culture was maintained on malt extract agar (MEA) slopes containing 2% malt extract, 2% agar at pH 5.6. The slant cultures were incubated at 30°C for 3-5 days until maximum sporulation. Sub-culturing was carried out every 2 weeks and frequently examined under a light microscope (FE6i, Olympus, Japan) to examine culture purity. The culture was stored at 4°C in a cold cabinet (MIR-153, Sanyo, Japan).

### Inoculum Preparation and Spore Count

Ten milliliter of sterilized 0.05% (w/v) diacetyl ester of sodium sulpho succinic acid (monoxal O.T.) was added to a slant culture of *P. notatum *NRRL-1249 having adequate growth. An inoculating wire-loop was used to disrupt the clumps of spores. The tube was shaken to obtain a uniform homogenous suspension. A haemocytometer was used to count the spore (A_560 nm _= 1) and found to be 1.5 × 10^6 ^CFU/ml.

### Fermentation Procedure

AXE production was carried out aseptically using solid state fermentation (SSF) in 250 ml Erlenmeyer flasks. Ten grams of wheat bran (dry basis) as the substrate with 4 cm depth was moistened by 10 ml of mineral salt solution (MSS) containing 0.1% KH_2_PO_4_, 0.05% KCl, 0.05% MgSO_4_.7H_2_O, 0.3% NaNO_3_, 0.001% FeSO_4_.2H_2_O and 0.1% (v/w) Tween-80 (pH 5.5) at a ratio of 1:1 (g:ml). The flasks were cotton plugged and sterilized in an autoclave (KT-40L, ALPCO, Midorigaoka, Hamara-Shi, Tokyo, Japan) at 103.5 kPa. pressure and 121°C temperature for 15 min. After sterilization, the medium was allowed to cool at room temperature and seeded with 1 ml of inoculum under aseptic conditions. The inoculum level was maintained during experiments with varying wheat bran mass. The flasks were incubated at 30°C for 48 h and shaken twice daily. All the fermentation experiments were run in a set of three replicates.

### Enzyme Extraction

After the required incubation period, 100 ml of distilled water was added into each of the flasks and agitated at 160 rpm in a rotary shaking incubator at 30°C for 1 h. The ingredients of the flasks were filtered and centrifuged at 8000 × *g *for 15 min.

### Analytical Techniques

The % constituent of wheat bran was determined using a solid-phase NIR spectrometer (SPQ-1243, Syngene, UK).

### Extracellular AXE Assay

The enzyme activity in the culture supernatant was determined following the modified method of Johnson *et al. *[9]. One unit of enzyme was defined as the amount of enzyme which releases 1 μmol *p*-nitrophenol per minute from 1 g of substrate under the defined assay conditions. For assay, 5 mM *p*-nitrophenyl-acetate dissolved in dimethyl sulfoxide was used as a substrate. Initially, 0.5 ml of acetate buffer (pH 5.8) and 0.5 ml of the appropriate enzyme dilution were added to the test tubes. To initiate the reaction, 1 ml of substrate was added. The reaction mixture was incubated at 30°C for 20 min. A blank was also run replacing 0.5 ml of the enzyme dilution with distilled water. A_420 nm _was determined using a double-beam UV/Vis scanning spectrophotometer (D-21496, Irmeco Gmbh, Heidelberg, Germany). Since the substrate may suffer non-enzymatic deesterification in alkaline pH, assays other than those in pH dependence studies were carried out at pH 5.8. The concentration of *p*-nitrophenol was calculated from the standard curve obtained under the assay conditions for enzymatic activity. The enzyme activity was expressed in U/g.

### Optimization of Significant Process Conditions

The level of wheat bran as a solid substrate was varied from 2.5-15 g and its effect on enzyme activity was studied. Various MSS levels (2.5-15 ml) as initial moisture content were also tested [10]. The pH of MSS was varied from 4.5 to 7. The time of incubation was ranged from 12-96 h after inoculation. The effect of nitrogen sources such as ammonium sulphate, urea, peptone and yeast extract was compared. Being optimal, the concentration of urea was varied from 0.1 to 0.6% (w/w). Later, the effect of Tween-80 (0.25-1.5%, v/w) on enzyme activity was also investigated [11].

### Statistical analysis and application of Plackett-Burman experimental design

Duncan's multiple range tests (Spss-16, version 9.5) were applied under one-way analysis of variance (I-ANOVA) and the treatment effects were compared after Snedecor and Cochran [12]. Significance was presented in the form of probability (*p*) values. The significant batch culture conditions affecting improved AXE productivity were identified using a 2-factorial system i.e., Plackett-Burman experimental design [13]. The variables were denoted at two widely spaced intervals and the effect of individual parameters on enzyme production was calculated by the following equations,(I)(II)

In Eq. I, E_ο _is the effect of first parameter under study while M+ and M- are responses of enzyme production by the fungal strain. N is the total number of optimizations. In Eq. II, E is the significant parameter, β_1 _is the linear coefficient, β_2 _the quadratic coefficient while β_3 _is the interaction coefficient among significant process parameters.

## Results and Discussion

**Figure 1:**
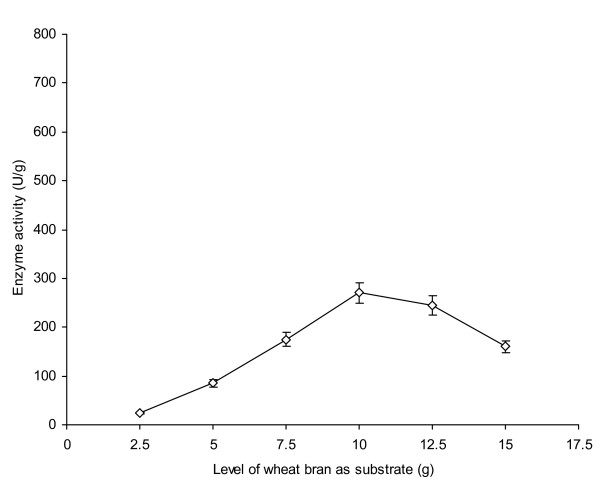
**Effect of various levels of wheat bran as a substrate on AXE production from *Penicillium notatum *NRRL-1249**. The substrate was moistened with MSS (pH 5.5) at a ratio of 1:1. The flasks were incubated at 30°C for 48 h. Y-bars show the standard deviation (±sd) among the triplicates.

Various agricultural by-products such as wheat bran, wheat straw, rice bran, rice straw, rice husk and corncobs could be used as substrate for microbial fermentation of both primary and secondary metabolites particularly xylanolytic enzymes [6,7]. The effect of different levels of wheat bran as a substrate was investigated on the production of an extracellular acetyl-xylan esterase by *Penicillium notatum *NRRL-1249 in solid state fermentation (SSF). An enzyme production of 25 U/g was obtained when 2.5 g of wheat bran with 1.2 cm depth was used (Figure 1). A gradual increase in enzyme production was observed (85-175 U/g) as the level of substrate was further increased from 5-7.5 g. Maximum enzyme production (270 U/g) was however, achieved with 10 g of substrate (4 cm bed height). It was due to the fact that optimal level of wheat bran provided an adequate amount of nutrients (1.32% proteins, 69% carbohydrates, 1.9% fats, 2.6% fiber, 1.8% ash content, 0.05% Ca, 0.17% Mg, 0.35% P, 0.45% K, 0.12% S and 0.23% various amino acids) required for the appropriate growth of microorganism. These nutrients were essential for microbial growth and subsequent secretion of xylanolytic enzymes as reported by Park [14]. The enzyme production was declined to 160 U/g at 15 g of the substrate (6.5 cm depth) possibly due to the thickening of medium or higher bed height, which gave hindrance in the proper aeration. It eventually resulted in the decreased air supply [4,7]. The sufficient supply of air is thus highly essential for better growth of mycelial hyphae as well as secretion of enzyme in the fermented mash-culture. Other workers also optimized wheat bran as the basal medium for AXE production [15].

**Figure 2:**
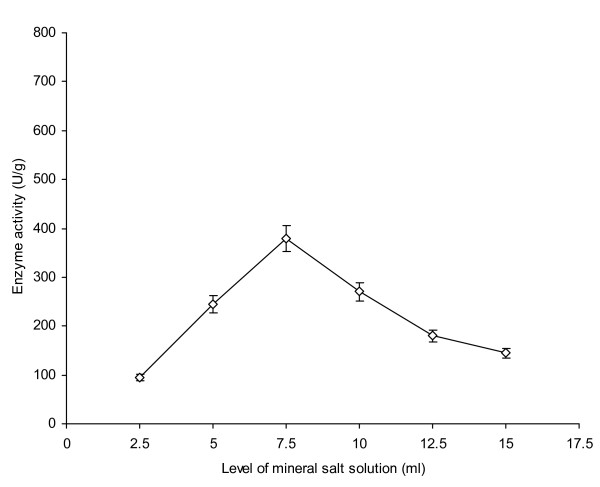
**Effect of different levels of mineral salt solution (pH 5.5) as initial moisture content on AXE production from *P. notatum *NRRL-1249**. Wheat bran (10 g) was used as a basal substrate and incubated at 30°C for 48 h. Y-bars show ± sd among the triplicates.

**Figure 3:**
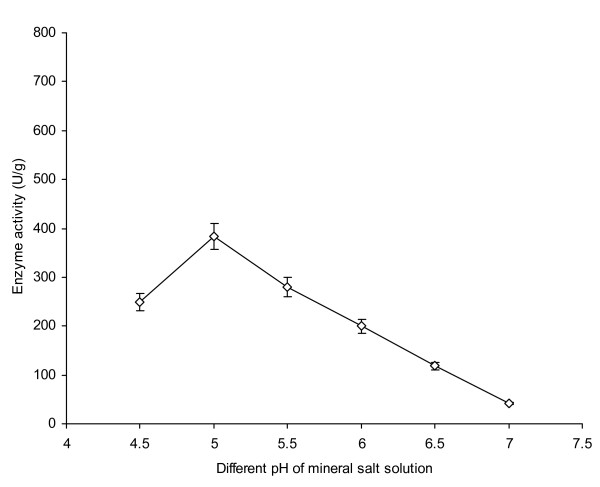
**Effect of different pH of mineral salt solution on AXE production from *P. notatum *NRRL-1249**. Wheat bran (10 g) was used as a basal substrate and moistened with MSS at a ratio of 1:0.75. The flasks were incubated at 30°C for 48 h. Y-bars show ± sd among the triplicates.

Initial moisture content has a profound effect on the secretion of metabolites [4]. Various levels of mineral salt solution (MSS, pH 5.5) were used to moisten 10 g of wheat bran as a solid substrate and their effect on AXE production by *P. notatum *NRRL-1249 was studied (Figure 2). The enzyme production remained almost insignificant from 2.5-5 ml of MSS as there was decreased solubility of nutrients observed which made the nutrients almost unavailable to the fungus causing a decline in its metabolic activity. Similar findings have also been reported by Haltrich *et al. *[16]. A significant increase (p ≤ 0.05) in the production rate was noticed (380 U/g) when 7.5 ml of the diluent was used. Beyond the optimal moisture level, enzyme production declined gradually (145 U/g at 15 ml of diluent). A higher level of moisture content is known to affect oxygen diffusion in the substrate which possibly caused water logging of the substrate. Extracellular pH has a strong influence on enzyme production as a number of microorganisms have a narrow optimal pH range at which they work progressively. Any deviation from this pH causes a decline in their enzyme production capability as reported by Linden [17]. The effect of initial pH on AXE production was also investigated (Figure 3). At pH 4.5, the enzyme production remained at 250 U/g. Low level of enzyme production at this pH level showed that the organism did not work well as pH started moving towards more acidic conditions. The organism was thus found to favor slightly acidic conditions but not sturdy acidic conditions substantiating the findings of Hamlyn *et al. *[4]. A better yield (384 U/g) was, however noticed when the pH of MSS was adjusted to 5. As the pH was increased up to 6, a marked decrease in enzyme production (200 U/g) was noticed. The pH optima of 5 have also been investigated for better enzyme production from other fungal cultures such as *Aspergillus *or *Streptomyces *spp. [18].

**Figure 4:**
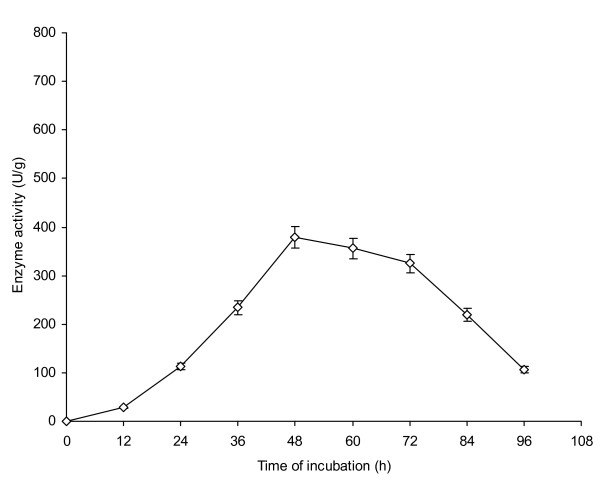
**Time of incubation for AXE production from *P. notatum *NRRL-1249**. Wheat bran (10 g) was used as a basal substrate and moistened with MSS (pH 5) at a ratio of 1:0.75. The flasks were incubated at 30°C. Y-bars show ± sd among the triplicates.

The rate of AXE production from *P. notatum *NRRL-1249 was also undertaken. Initially at 12 h of incubation, 28 U/g of enzyme activity was achieved (Figure 4). Enzyme production in the fermented mash increased gradually with the increase in incubation period from 24-36 h. However, the optimal results (379 U/g) in terms of enzyme production were achieved at an incubation period of 48 h. Afterwards, the enzyme production declined gradually (60-84 h), becoming very low 106 U/g at 96 h of incubation. It was due to the decreased amount of available nutrients in the production medium, age of fungi, the presence of inhibitors produced by the culture or depletion of sugar contents as reported by Kansoh and Gammal [19]. Judith and Junior [20] obtained optimal enzyme activity 144 h after inoculation; therefore present work is economically more significant because reduction in incubation period could reduce the cost of enzyme production. Similar kinds of finding have also been reported previously [21].

**Figure 5:**
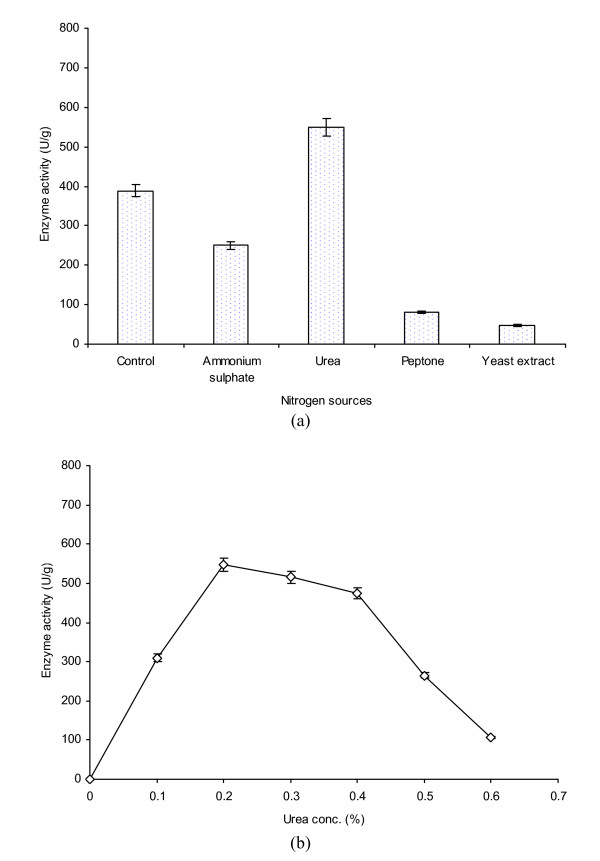
**Evaluation of, a) various nitrogen sources added at a level of 0.2%, and b) urea concentrations on AXE production from *P. notatum *NRRL-1249**. Wheat bran (10 g) was used as a basal substrate and moistened with MSS (pH 5) at a ratio of 1:0.75. The flasks were incubated at 30°C for 48 h. In the control MSS, 0.2% magnesium sulphate was used as a nitrogen source. Y-bars show ± sd among the triplicates.

In the present study, various nitrogen sources including ammonium sulphate, urea, peptone and yeast extract were compared at a level of 0.2% for AXE production from *P. notatum *NRRL-1249. The results were compared with the control (having 0.2% magnesium sulphate). The control gave 388 U/g of enzyme activity (Figure 5a). The effect of ammonium sulphate on enzyme activity was not found encouraging (p ≤ 0.05). The enzyme activity was found to be the highest (550 U/g) with urea as a sole nitrogen source. Thus, 1.42 fold better activity was achieved compared to the control. Peptone and yeast extract, however exhibited the least activity i.e., 80 and 47 U/g, respectively. Similar kinds of findings have also been reported by Ximenes *et al. *[22]. The effect of different levels of urea on the enhanced AXE production was also studied (Figure 5b). The level was varied from 0.1 to 0.6% (w/w) for separate trials. At 0.1% level of urea, enzyme activity was found to be 310 U/g. However, the maximum enzyme activity (548 U/g) was achieved at 0.2% of urea as a sole nitrogen source. There was a marked decline in the activity (106-265 U/g) when higher levels (0.5-0.6%) were employed. It was due to the fact that higher concentration of free nitrogen caused toxicity which had adverse effects on the development of biomass and enzyme productivity. The work is substantiated with the findings reported earlier [15,23].

**Figure 6:**
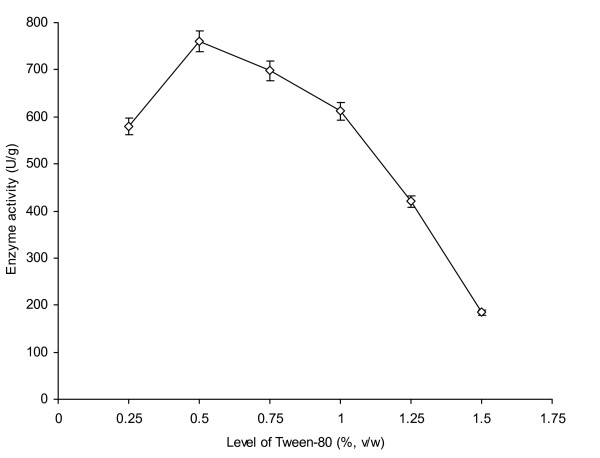
**Effect of different levels of Tween-80 on AXE production from *P. notatum *NRRL-1249**. Wheat bran (10 g) was used as a basal substrate and moistened with MSS (pH 5) at a ratio of 1:0.75. The flasks were incubated at 30°C for 48 h. Y-bars show ± sd among the triplicates. Each mean value differ significantly at a level of p ≤ 0.05.

Polyoxyethylene sorbitane monooleate (Tween 80) in an appropriate level may have a strong effect on the efficiency of fermentation medium to produce higher level of AXE [9]. The effect of different levels of Tween-80 was investigated on the production of enzyme from *P. notatum *NRRL-1249 (Figure 6). At 0.25% level of Tween-80, enzyme activity was noted to be 580 U/g. However, the maximum enzyme activity (760 U/g) was achieved at 0.5% Tween-80 which was over 35% improvement in net enzyme activity. It was due to the fact that Tween 80, being a complex source of nutrients, had a strong effect on enzyme synthesizing capability of producer organism [24]. There was a marked decline in the activity when higher levels of Tween-80 were used which significantly (p ≤ 0.05) reduced at 1.5%. It was attributed to the over growth of fungal culture and exhaustion of essential minerals from the fermented mash-culture as reported by Belancic *et al. *[25]; Knob and Carmona [26].

The 2-factorial experimental system i.e., Plackett-Burman design was applied to determine the significant process parameters involved in AXE production by *P. notatum *NRRL-1249 (Table 1). The validation of the model was investigated under the conditions predicted against the responses obtained for enzyme production. A differential correlation was noted between the observed and predicted values as reported by Burkert et al. [27]. The optimal levels of the parameters for improved enzyme production under SSF were incubation period (48 h), initial pH (5), 0.2% (w/w) urea as nitrogen source and 0.5% (v/w) Tween-80 as a stimulator. The optimal level of wheat bran as a substrate was found to be 10 g. The statistical analyses of the responses for AXE production were also performed (Table 2). The correlation (0.122*E*+0005) of A, B, C and D for E values depicted that the model was highly significant (p ≤ 0.05). Correspondingly, the lower probability values indicated that the model terms are valid. The analysis of linear, quadratic and interaction coefficients were performed on the batch culture results which highlighted that enzyme production was a function of the independent parameters [13]. The addition of MSS as a diluent (degree of freedom 3) was found necessary for maintaining the possible spatial conformation of enzyme and thus have an important physiological role in the enzyme activity. According to these results, the fungal strain of *P. notatum *NRRL-1249 could be considered as an organism of choice for AXE productivity.

**Table 1:** Application of 2-factorial design at various process parameters for AXE production by *P. notatum *NRRL-1249*

Process parameters at 2-factorial design					AXE production (U/g)	
**Wheat bran (g)**^**A**^	**MSS pH**^**B**^	**Time of incubation (h)**^**C**^	**Urea (%, w/w)**^**D**^	**Tween-80 (%, v/w)**^**E**^	**Observed**	**Predicted**

5	4.5	24	na	na	185	198
7.5	4.5	36	na	na	312	385
10	5	36	0.1	0.25	455	560
10	5	48	0.2	0.5	760	832
12.5	5.5	60	0.3	0.75	646	811

*The different letters represent significant process parameters for AXE production. Statistical analysis of the model was based on 2-factorial experimental design. 'na' means that nitrogen source was not added.

**Table 2:** Comparison of statistical analysis of significance level and probability values for AXE production*

Significant process parameters	Sum mean values	F-value	Degree of freedom (df)	Probability < p >
A	188	12.34	1	0.0874
B	315	25.48	1	0.0762
C	446	28.15	2	0.0595
D	781	30.16	3	0.0238
E	508	22.15	2	0.0316
Correlation	0.122*E*+0005			

*The letters represent significant process parameters (level of wheat bran, pH of MSS, incubation period, urea conc. and level of Tween-80) for AXE production. CM - 16.42; *R*^*2 *^- 0.128.

## Conclusion

In the present study, *Penicillium notatum *NRRL-1249 was used for AXE production in solid state fermentation. Wheat bran (10 g) was employed as the basal carbon source with 4 cm bed height. Mineral salt solution (MSS, 7.5 ml) containing 0.1% KH_2_PO_4_, 0.05% KCl, 0.05% MgSO_4_.7H_2_O, 0.3% NaNO_3_, 0.001% FeSO_4_.2H_2_O and 0.1 ml Tween-80 at pH 5 as an initial moisture content was also optimized. The most notable finding was the addition of 0.5% (v/w) Tween-80 as an enzyme stimulator in the fermented mash-culture which supported a maximal of 760 U/g enzyme activity. An overall improvement of more than 35% in terms of enzyme activity was accomplished when the significant process parameters were determined after Plackett-Burman design. The value of AXE correlation (0.122*E*+0005) depicted that the model terms are highly significant (*HS*, p ≤ 0.05) indicating commercial utility of the fungal culture (df = 3, LSD = 0.126). However, enzyme characterization is a pre-requisite prior to scale up studies.

## Abbreviations

SSF: Solid state fermentation; MSS: mineral salt solution; MEA: malt extract agar; NRRL: northern regional research laboratories; U/g: units per gram; rpm: revolutions per minute; HS: highly significant; <p>: probability of significance level.

## Competing interests

The authors declare that they have no competing interests.

## Authors' contributions

SAt and SAl conceived the study and performed the experimental work. MNA and HI supervised and provided necessary facilities. Authors read and agreed to the final manuscript.
